# Bilingual Cognitive Control in Language Switching: An fMRI Study of English-Chinese Late Bilinguals

**DOI:** 10.1371/journal.pone.0106468

**Published:** 2014-09-02

**Authors:** Hengfen Ma, Jiehui Hu, Jie Xi, Wen Shen, Jianqiao Ge, Feng Geng, Yuntao Wu, Jinjin Guo, Dezhong Yao

**Affiliations:** 1 School of Foreign Languages, Civil Aviation University of China, Tianjin, China; 2 Key Laboratory for NeuroInformation of Ministry of Education, School of Life Science and Technology, University of Electronic Science and Technology of China, Chengdu, China; 3 School of Foreign Languages, University of Electronic Science and Technology of China, Chengdu, China; 4 Key Laboratory of Behavioral Science, Institute of Psychology, Chinese Academy of Sciences, Beijing, China; 5 Department of Radiology, Tianjin First Center Hospital, Tianjin, China; 6 Center for MRI Research, Academy for Advanced Interdisciplinary Studies, Peking University, Beijing, China; University Children’s Hospital Tuebingen, Germany

## Abstract

The present study explored the bilingual cognitive control mechanism by comparing Chinese-English bilinguals’ language switching in a blocked picture naming paradigm against three baseline conditions, namely the control condition (a fixation cross, low-level baseline), single L1 production (Chinese naming, high-level baseline), and single L2 production (English naming, high-level baseline). Different activation patterns were observed for language switching against different baseline conditions. These results indicate that different script bilingual language control involves a fronto-parietal-subcortical network that extends to the precentral gyrus, the Supplementary Motor Area, the Supra Marginal Gyrus, and the fusiform. The different neural correlates identified across different comparisons supported that bilingual language switching involves high-level cognitive processes that are not specific to language processing. Future studies adopting a network approach are crucial in identifying the functional connectivity among regions subserving language control.

## Introduction

Speaking two languages is becoming the normal rather than odd in the modern world. An interesting question is what control mechanisms are engaged to allow bilinguals to switch across languages smoothly [1], [2]. Cognitive control mechanism is crucially important for bilinguals in the selection and temporal sequencing of different language representations. This mechanism enables bilinguals to select the lexical item in the target language while inhibiting the non-target language [3], [4]. Then a further question is how this language switching expertise relates to the domain-general cognitive control ability [5].

To account for the bilingual language switch mechanism, Abutalebi and Green [6] proposed a neurocognitive model consisting of five brain regions: left dorsolateral prefrontal cortex (DLPFC), anterior cingulate cortex (ACC), caudate nucleus, and bilateral supramarginal gyri (SMG). It is notable that these areas are also involved in cognitive control in general. For example, the left DLPFC and bilateral SMG (part of the inferior parietal lobule) are part of a fronto-parietal network of attention [7]. The ACC is associated with error detection and is part of the cognitive network that allocates neural resources to guide behaviour [8]. The caudate is important in mediating cortical activation in the ACC and prefrontal regions to enhance switching the focus of attention between stimulus representations [9].

In contrast to this fronto-parietal-subcortical network, Luk et al. [10] pulled out eight regions showing significant and reliable activation in language switching relative to single language production in a quantitative meta-analysis of multiple researches. These include the left IFG, left middle temporal gyrus (MTG), left middle frontal gyrus (MFG), right precentral gyrus, right STG, midline pre-supplementary motor area (pre-SMA), and bilateral caudate nuclei. Compared with Abutalebi and Green model [6], Luk et al. [10] identified no activation in the ACC and bilateral SMG, but identified additional clusters in the pre-SMA, left MTG and the right precentral gyrus. Luk et al. attributed the different activation patterns to the nature of the baseline tasks. Language switching may induce different activations against different levels of baseline, namely, a low-level baseline (i.e., a fixation cross with no language production task), a high-level baseline (i.e., L1 or L2 single language production) [10]. For example, the bilateral SMG may be involved in the general network of attention and phonological processing, but not be differentially activated during switching. Thus, an activation of SMG might be derived from contrasts between language switching and the low-level baseline condition. Meanwhile, the same activation might not be observed from contrasts between language switching and high-level baseline conditions.

Thus, the present study set out to examine the bilingual cognitive control mechanism by comparing Chinese-English bilinguals’ language switching in a blocked picture naming paradigm against three baseline conditions, namely the control condition (a fixation cross, low-level baseline), single L1 production (Chinese naming, high-level baseline), and single L2 production (English picture naming, high-level baseline). We are interested in whether the fronto-parietal-subcortical network involving ACC and SMG would be observed for the language switching relative to the high-level baseline condition. These results would help to examine how the neurocognitive model of bilingual language switching proposed in Abutalebi & Green [11], [12] and Luk et al. [10] may be related to cognitive control in general.

In a typical picture naming paradigm, Wang et al [13] investigated the neural substrates of language switching with Chinese English bilinguals. They found that compared to the L1 and L2 single naming conditions, language switching induced greater activations in the right superior PFC, left middle and superior frontal cortex, and right middle cingulum and caudate. These areas were different from Hernandez et al. [14]–[16] findings with Spanish–English bilinguals, which showed increased activity in the DLPFC, precentral gyrus, SMA and the superior parietal lobule for language switching against single language naming. The difference might be attributed to several reasons, one of which is that Hernandez et al. [14]–[16] used a blocked fMRI design, whereas Wang et al. [13] adopted an event-related design. Wang et al. [17] further compared Chinese English bilinguals’ sustained (block-based language switching) and transient (trial-based language switching) language control with blocked and event-related design respectively in a digit naming task. They found more activation in the bilateral inferior frontal, middle prefrontal and frontal gyri for sustained bilingual control than single language, whereas transient bilingual control activated the left inferior and superior parietal lobule, MFG, and the right superior parietal cortex. These results are very significant in showing that sustained and transient bilingual control might involve different neural patterns. Yet, neither condition induced activation in the ACC, caudate and SMG, which were shown to be important for language control [12].

Interestingly, with a similar blocked picture naming paradigm, Guo et al [18] detected more activations in dorsal ACC and SMA for language switching (mixed naming block) against single language naming. However, they found no activation in the left caudate and SMG, which have been shown to be important for cognitive control with same script bilinguals [6], [12], [14]. Guo et al. [18] speculated it might it might be easier to switch between different script languages such as Chinese and English than same-script languages such as Spanish and English. However, both SMG and caudate were observed in the recent Hosoda et al. [19] study with Japanese English bilinguals. They found that relative to backward switching, transient forward switching (L1 to L2) induced stronger activities in the right PFC, left STG/SMG, ACC, left IFG, and caudate nucleus. Thus, more data are needed to map the terrain for language control mechanisms of different script bilinguals.

To this aim, we tested the late Chinese-English bilinguals’ language production in a picture naming paradigm with block-based fMRI technique. Due to the inconsistent activation patterns emerged in different script bilingual language production, we were especially interested the activation of the ACC, SMG and caudate structures in language switching against three different baseline conditions (fixation cross, single L1 naming, single L2 naming).

## Methods

### Subjects

Twenty-two adult bilinguals (five female and seventeen male) with a mean age of 22.6 (SD = 3.75, Range 20–25) participated in the current experiment. None had any history of neurological or psychiatric disorders. All were Chinese–English unbalanced bilinguals who started to learn English upon entering junior high school at about 12 years old. Participants are all studying English as their major in the school of foreign languages in the Civil Aviation University of China. They have passed the Test for English Majors band 8, which is equivalent to a TOEFL iBT 100 or IELTS band 7.5. All were right handed as assessed by handedness questionnaire. Written informed consent was obtained from all participants according to protocols approved by the Ethical Committee of Civil Aviation University of China before commencing all experimental procedures.

### Materials

A total of 60 simple black-white line graph picture stimuli were used during behavioral and fMRI sessions. The word length of these pictures were controlled (2 syllables for Chinese and mono- or bi-syllables in English). Their Chinese and English equivalent names have comparable frequency (67.4 vs. 74.1 wpm) and rated imageability (5.8 vs. 6.1 on an 8 point scale). Ten additional pictures were used in the practice session. No cognates were present for both behavioral and experimental stimuli.

### Experimental procedure

Block-based fMRI design was used in the present study. Altogether there were 25 blocks, each lasting 20 s with ten trials (TR = 2). Experimental blocks were assigned into three conditions (see Table 1 for an illustration): language switching, L1 naming (high-level baseline) and L2 naming (high-level baseline). The control block (low-level baseline) contained ten trials of fixation cross. There were altogether 12 experimental blocks and 13 control blocks. These blocks were divided into two sessions, each lasting 8 min and 20 s. All participants took part in two scanning sessions in a random order. The order of the blocks in each session was pseudo-randomized. There was always a control block in between each experimental block to temper carry over effects between L1 and L2.

**Table 1:** The three experimental conditions and the corresponding stimuli.

Picture	A GUITAR	A MOUSE	PANTS	…
Condition	“Cue”→response	“Cue”→response	“Cue”→response	…
Switching	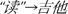	*“read”→mouse*	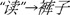	…
L1 naming	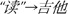	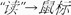	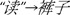	…
L2 naming	*“read”→guitar*	*“read”→ mouse*	*“read”→pants*	…

To minimize movement artifacts of overt naming, we adopted the covert naming task, as has been done in several major studies on language switch [14], [20]. During the experiment, subjects were asked to covertly name pictures according to the visual cue “read” (name the picture in English) or “
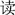
” (name the picture in Chinese). The visual cue was presented for 200 ms first, then followed by a picture for 1800 ms. For each trial in the control block, a small “+” was presented for 200 ms followed by a large “+” for 1800 ms. Subjects were asked to fixate their eyes on the cross silently and no response was required. Before the experiment, subjects first learned Chinese and English names of all pictures and did practice tasks similar to experimental tasks.

As participants made no overt verbal responses to the pictures in the scanner, behavioral data were collected again outside of the scanner about 1 month later in a quiet, isolated room with the same group of participants.

### Procedure

Functional imaging was performed with a General Electric 3.0 Tesla magnetic imager equipped with echo-planar imaging (EPI) from Advanced NMR (Wilmington, MA). For each subject, a conventional sagittal scout scan was obtained in order to determine the slices used during functional imaging. Using an EPI gradient echo sequence (TR = 2000 ms; TE = 30 ms; a 64*64 scan matrix with a 24 cm FOV) 176 images were obtained for each subject over 33 slices (3 mm thick/1 mm gap). The most inferior and superior slices approximately corresponded to z = −24 and z = +65, respectively. A set of coplanar high-resolution EPI structural images was also collected at the same time and used to spatially normalize each subject’s data into a standardized template. Behavioral testing was conducted for each subject after the fMRI sessions.

### Data analysis

The functional images for each subject were preprocessed and analyzed using SPM5 [21] which includes realignment, spatial transformation and smoothing using a 9 mm FWHM isotropic Gaussian kernel to increase the signal-to-noise ratio. Statistical random effects analyses were also conducted using SPM5 (Welcome Department of Cognitive Neurology, Institute of Neurology, London, UK). Images were corrected for height using an uncorrected threshold value of p<0.001 and contained at least 10 contiguous voxels. Statistically significant areas were superimposed on individual brain anatomy in MNI space using SPM routines. Direct comparisons were also exclusively masked by activation vs. rest contrasts which were thresholded at p<0.001. The masking procedure was employed in order to eliminate any voxels in which the condition of interest would be less active than the rest condition. Figures were created using the brain software and a template brain from Alan Evans of the Montreal Neurological Institute [22].

## Results

### Behavioral results

Behavioral data from the delayed post-test were placed into a response language (L1 vs L2)* block type (single/mixed) repeated-measures within-subject ANOVA. For the response accuracy, results revealed a main effect of the response language, F(1,32) = 9.40, p = 0.004. The effect of block type was not significant, p = 0.36, nor was their interaction, p = 0.71. This showed that the participants were more accurate in L1 Chinese naming than in L2 English in both single language and mixed naming block. Trials with errors were then excluded from further analyses on the response time. ANOVA results for the response time showed a main effect of the response language, F(1,32) = 10.29, p = 0.003. The main effect of block type was also significant, F(1,32) = 8.78, p = 0.006. The interaction between response language and block type was not significant, p = 0.16. This result suggested that naming pictures in L1 was faster than in L2 and naming in single language is faster than naming in the mixed condition.

### Neuroimaging results

**Figure 1:**
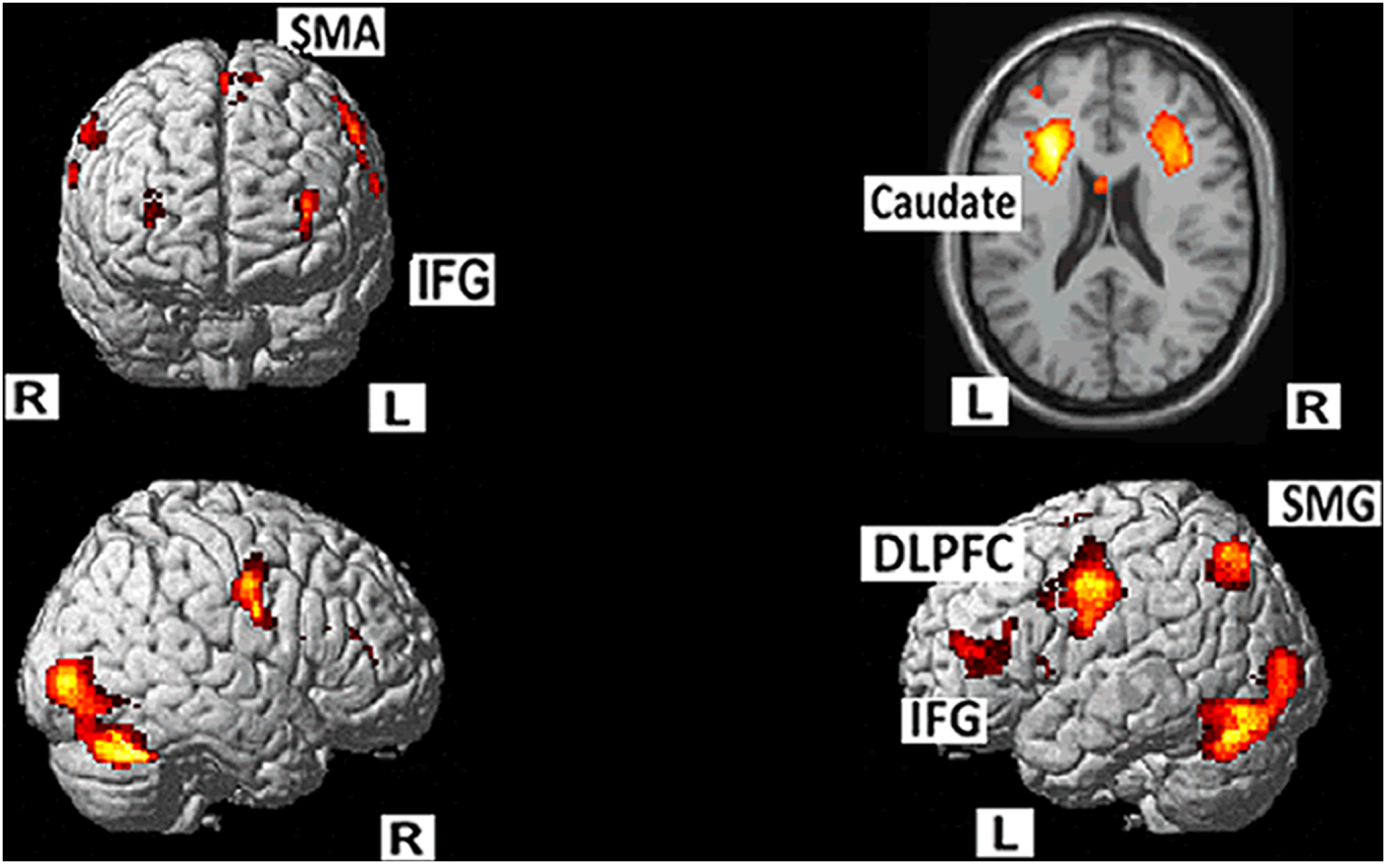
Mixed against control condition showed increased activity in a network of areas including the left IFG, the precentral gyrus, SMA, the left IPG, the bilateral fusiform gyrus extending to the occipital lobe, as well as the subcortical regions including the left caudate, and the right hippocampus.

Direct comparisons were conducted between the mixed condition (language switching) and the other three conditions, namely, control condition (fixation cross, low-level baseline), L1 naming (single Chinese production, high-level baseline), L2 naming (single English, high-level baseline). Mixed versus control condition comparisons revealed increased activity in a network of areas including the left inferior frontal gyrus (IFG), the precentral gyrus, SMA, the left inferior parietal gyrus (IPG), the bilateral fusiform gyrus extending to the occipital lobe, as well as the subcortical regions including the left caudate, and the right hippocampus (Table 2, Figure 1). These results replicated and extended previous findings on different script language switching.

**Table 2:** Areas of increased activity for the mixed vs. control condition (MNI coordinates).

Brain Region (positive)	BA	Voxels	Peak t-value	x	y	z
L. Precentral/SMA/IFG	6/4/9/32	4403	8.36	–30	–4	36
L. Inf Parietal G/Mid Occipital	7	663	7.05	–28	–68	46
L Fusiform	19/37	1538	5.37	–36	–28	–20
L. Caudate		123	4.85	–24	–40	12
R. Precentral/Inf Front G/Insula	6	2126	6.06	20	8	38
R. Cerebellum/Fusiform/Inf Occipital	19/37	1939	8.10	40	56	–28
R. Hippocampus		196	5.22	34	–38	6

**Figure 2:**
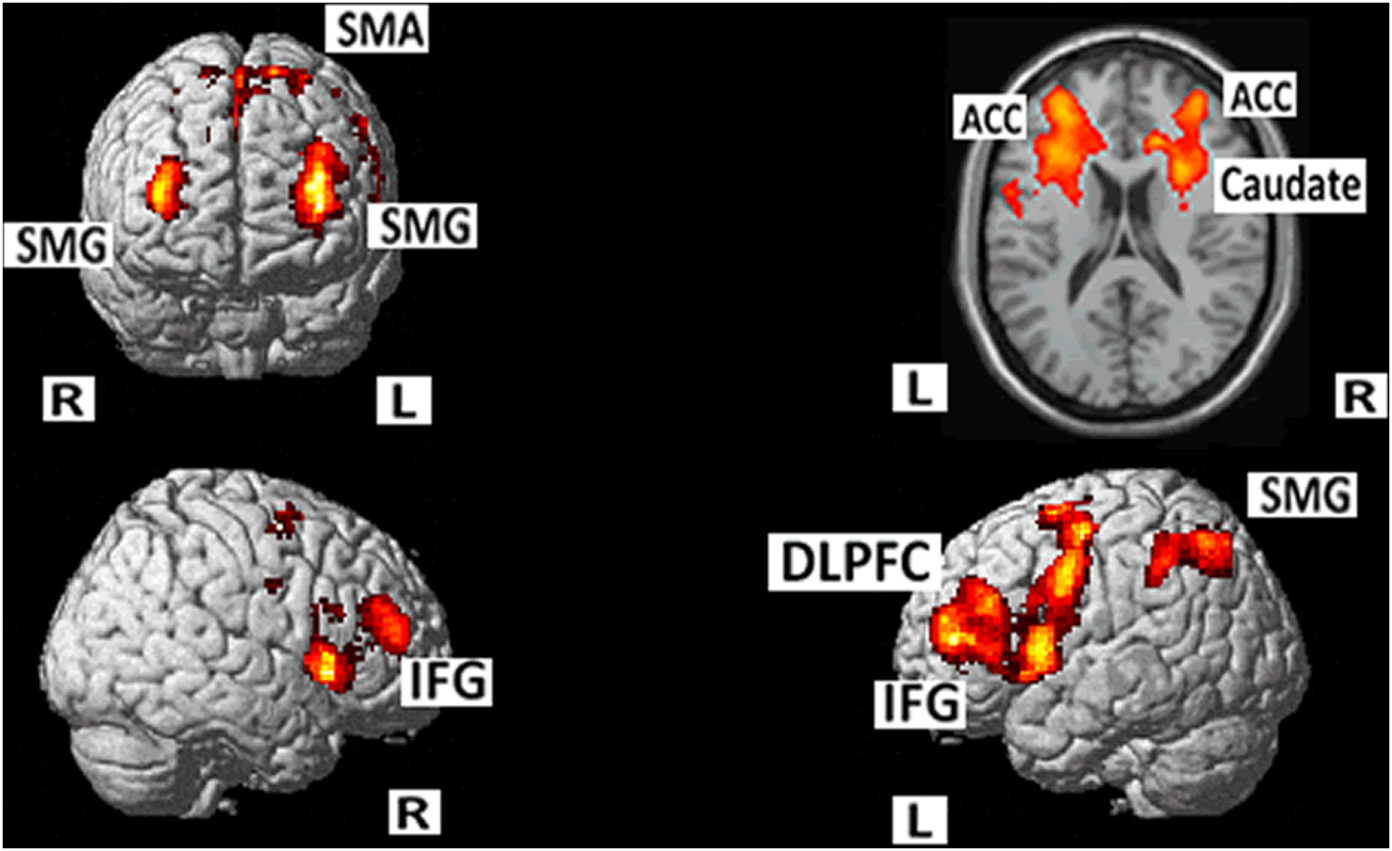
Mixed against L1 Chinese naming condition showed broadly distributed areas falling into two big clusters. One peaked at the posterior part of the left IFG and extended all over the prefrontal cortex and to the bilateral SMA, left insula, left putamen and right caudate. The other peaked at the left inferior parietal gyrus and extended to the SMG and the Angular and precuneus. (cluster-level FWE corrected, p<0.05).

Language switch against single L1 Chinese naming showed broadly distributed areas falling into two big clusters. The first one peaked at the posterior part of the left IFG and extended all over the prefrontal cortex and to the bilateral SMA, left insula, and basal ganglia structures including left putamen and right caudate. The second big cluster peaked at the left inferior parietal gyrus and extended to the left Supramarginal gyrus (SMG) and the Angular and precuneus (Table 3, Figure 2).

**Table 3:** Areas of increased activity for the mixed condition vs. blocked naming in Chinese (MNI coordinates).

Brain Region (positive)	BA	Voxels	Peak t-value	x	y	z
L. Inf Front G/Supp Motor Area (extending to)	6/9/44/45/47	15396	9.37	–32	42	10
L Prefrontal/Precentral/Insula/ACC/putamen						
R Inf Front G/Supp Motor Area/ACC/Caudate						
L. Inf/Sup Parietal G (extending to)	7/19/39	1232	6.26	–28	–64	46
L. SMG/Angular/Precuneus	39					

**Figure 3:**
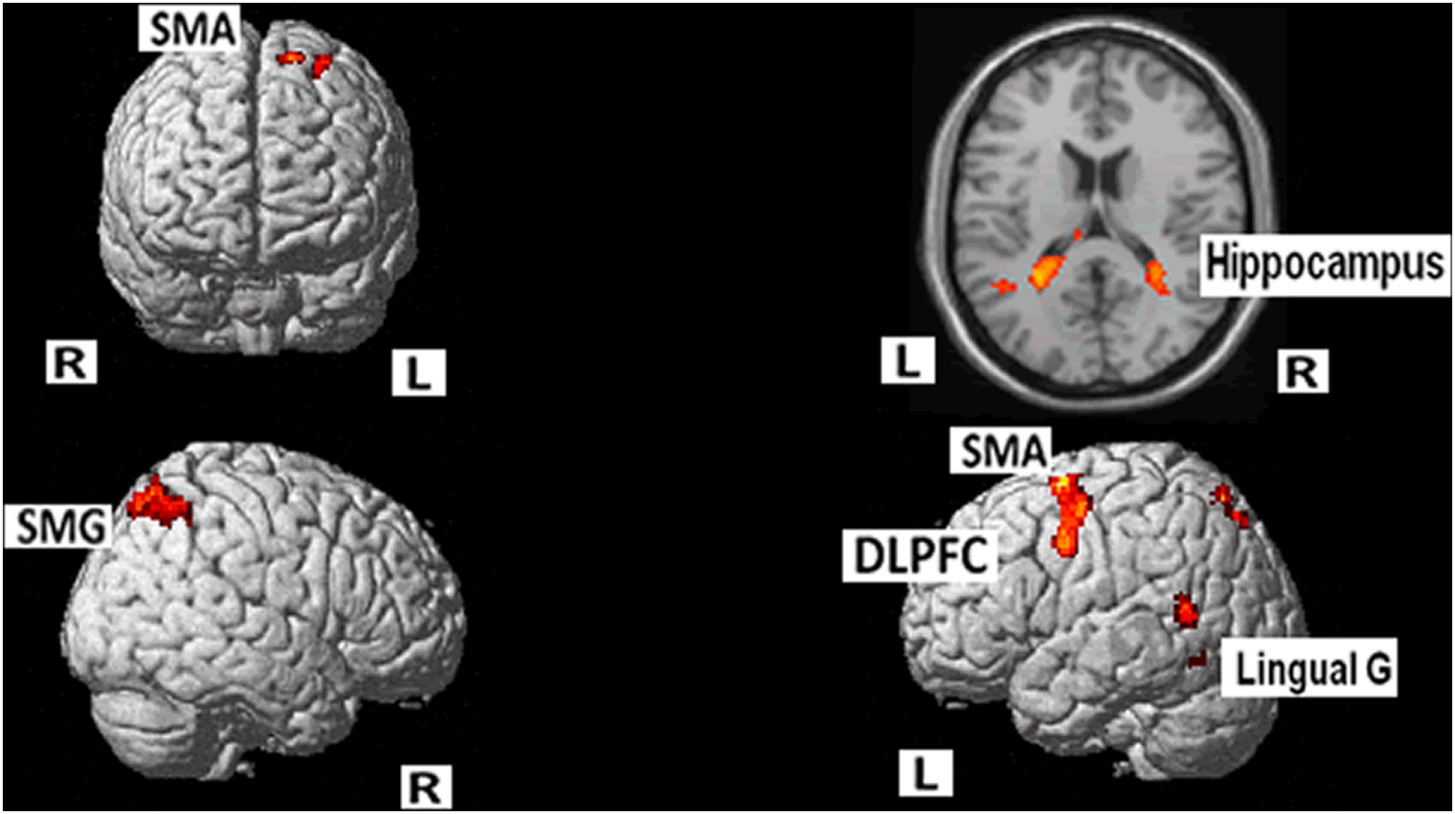
Language switching against L2 English naming condition showed activations in the left IFG, the bilateral Precentral gyrus and SMA, the bilateral IPG, the bilateral fusiform, the left lingual gyrus, the left inferior temporal gyrus, and the bilateral hippocampus. (cluster-level FWE corrected, p<0.05).

However, the language switching (mixed condition) against single L2 English naming condition induced activations mainly in the left IFG, the bilateral Precentral gyrus and SMA, the bilateral IPG, the bilateral fusiform, the left lingual gyrus, the left inferior temporal gyrus, as well as the bilateral hippocampus (Table 4, Figure 3). Notably, the caudate did not emerge in this comparison. We will refer to this in the discussion.

**Table 4:** Areas of increased activity for the mixed condition vs. blocked naming in English (MNI coordinates).

Brain Region (positive)	BA	Voxels	Peak t-value	x	y	z
L. Sup Front G/SMA	6	751	5.40	–44	0	40
L IFG/Mid Front	9	172	4.53	–38	42	38
L. Fusiform/Lingual G/Hippocampus	19/39	1342	7.06	–8	–26	22
L. Inf Parietal	7/40	242	4.61	–40	–52	54
L Precuneus/Sup Parietal	7	322	5.39	–10	–68	56
R. Inf Parietal/Angular	7/40	659	5.64	24	–56	42
R. Fusiform/Hippocampus/Precuneus	19	1059	8.59	32	–46	2
R Precentral/SMA	6/9	159	4.41	22	–6	52

## Discussion

The present study examined different script bilinguals’ cognitive control mechanism in language switching against three baseline conditions, namely the control condition (low-level baseline), the L1 Chinese naming (high-level baseline) and L2 English naming (high-level baseline). Results showed both similarities and differences in activations for language switching against three baseline conditions. These regions replicated and extended previous findings on the neural correlates of bilingual cognitive control in different script language switching with Chinese-English [13], [17], [18] and Japanese-English [19], [23] subjects. Below we discuss these activations in the fronto-parietal-subcortical network for language switching by the Abutalebi and Green [6] and Luk et al. [10]. The Abutalebi and Green [6] model features five brain regions at work: left DLPFC, ACC, caudate nucleus, and bilateral SMG. Luk et al. [10] model supports the role of left PFC and caudate, but fails to find the ACC and bilateral SMG. Additionally, they find activations in pre-SMA, the right precentral gyrus, and the left middle temporal gyrus. We consider the similarities and differences between the present results and the bilingual language switch models in turn.

First of all, language switching in the mixed condition induced frontal activations against all three baseline conditions. Yet, the present results showed that the frontal activation patterns were different across different comparisons. Relative to the control (fixation cross) and L1 naming conditions, language switching evoked broad activations in bilateral inferior frontal, and DLPFC areas. However, only the posterior part of the left DLPFC was activated for the language switching against L2 English naming block. This robust effect for left DLPFC across all three comparisons provided further evidence that this area might be a key mediator in cognitive control of bilingual switching. The prefrontal cortex is involved in decision-making, working memory, response selection and inhibition, thus it might work together with the ACC and the basal ganglia to inhibit interferences from the non-target language [24]. This result is also in line with the series of fMRI studies with different Spanish-English bilinguals [14], [24], German-English bilinguals [25], Dutch–French bilinguals [26], suggesting that the left prefrontal cortex is consistently implicated in the mechanism of language switching and selection.

The present study also revealed robust parietal activation for language switching against all three baseline conditions. These activations typically fell into the SMG area specified in the Abutalebi and Green model [6]. This result is in line with Hosoda et al. [19] in showing that SMG is involved in bilingual language control actively [6], [12], [14]. However, this structure was not detected in Wang et al. [17] and Guo et al [18]. Luk et al. [10] attributed lack of activation in SMG to the nature of the high-level baseline tasks, which all required phonological processing. The present results showed that bilateral SMG was more activated in bilingual language switching despite its general role in language processing. As noted in Abutalebi and Green [6], the parietal cortex is involved in working memory and maintenance of task representations. This might be achieved through parallel networks, one directly linking the posterior parietal cortex to the prefrontal cortex, and one passing through the caudate nucleus.

The caudate activation has been consistently reported in language switching with different script bilinguals [13], [19]. However Guo et al. [18] reported no caudate activation for language switching against single language production in a block-based picture naming task. The current results showed that the different results in the previous literature might be related to the baseline condition. In the present study, the caudate was activated for the mixed condition against the control and L1 naming condition, but not for the mixed condition against L2 English naming condition. The caudate nucleus, and the basal ganglia in general, have been shown to play a role in mediating cortical activation in the ACC and prefrontal regions to enhance switching the focus of attention between stimulus representations [9]. Damage to the basal ganglia led to disruption in late integrational language processing [27], [28]. Thus, activation of the caudate was consistenly found in L2 word production when subjects have to allocate more neural resources to inhibit the L1 [29]. Therefore, the present results actually showed that the caudate was involved in inhibiting the non-target language during language switching and L2 English naming condition. The caudate related basal ganglia structures are ideal candidates for inhibition function because of their forward and backwards connections to the prefrontal cortex. Engagement of striatal neurons by top-down signals would allow the striatum to enhance and suppress particular sets of representations through prefrontal-basal ganglia loops [30]–[33]. Consist with this, frontal areas, especially the right inferior frontal gyrus, has been shown to be linked to inhibition in studies on language switching with both Japanese and English bilinguals [19] and Dutch-English-German trilinguals [34].

The present study also showed robust activation of precentral gyrus, SMA for language switching relative to both control and single language production conditions. Although these motor areas were not implicated in the bilingual control network of Abutalebi and Green [6], they emerged in the meta-analysis of Luk et al. [10]. Importantly, the activation of precentral gyrus and SMA have been consistently reported in Chinese-English bilingual word production tasks [17], [18], [29], suggesting that these areas might be actively recruited to resolve the interference during phonological retrieval across languages, especially in the case of different script bilingual production [35]–[37].

The ACC is closely related to the SMA. Highly involved in error monitoring and detection, the ACC has been found to show less activity during correct trials and more activity during error trials [38], [39], thus ACC was implicated to signal to the prefrontal cortex to bias against incorrect selection when an erroneous language was chosen. Luk et al. suggested that the SMA related structures may sometimes combine with the dorsal ACC to form a region known as the Rostral Cingulate Zone [40], [41] in performing demanding tasks in terms of response control, performance monitoring and error detection [42], [43] and it is the superior part of the Rostral Cingulate Zone that is consistently activated in language switching [10]. Thus, the robust activation of the SMA for language switch against all three baselines in the present study might suggest a role of the SMA in serving as a control tradeoff of the ACC [10], [29], [44]. Consistent with this explanation, the present study replicated Guo et al [18] in finding robust ACC activation for language switching against Chinese naming condition only, but not for language switching against the other two baseline comparisons.

The current results also showed activation in bilateral fusiform, the left lingual gyrus for the switching condition relative to single English production. These areas have been found in studies with picture naming in L2, showing that L2 production involves stronger demands than L1 due to more controlled articulation and visual form processing [12], [14], [29]. As the modulation of the lingual gyrus and fusiform Gyrus was consistently reported in different script bilinguals [18], it might suggest that in language switching, additional cognitive control is needed in order to establish a link between an object representation and its corresponding articulatory code. Further tests are needed to identify the specific roles of these structures in lexical selection and inhibition. The present results additionally showed increased right hippocampus activation for language switching against control condition, but bilateral hippocampus activation for switching against L2 English production. Hernandez [14] argued that L1 picture naming might be associated with more robust word meaning retrieval while L2 picture naming might be associated with increased reliance on recognition memory. How the hippocampus contributes to language switching mechanism deserves future investigation.

To summarize, the present study strongly supported that different script bilingual cognitive control involves a fronto-parietal-subcortical network that extends to the precentral gyrus, the SMA, SMG and bilateral fusiform. A full exploration of the structures involved requires the comparison of bilingual language switching against different levels of baseline condition. The different neural correlates identified across different comparisons also supported that bilingual language switching involves high-level cognitive processes that are not specific to language processing. Future studies adopting a network approach [45]–[47] are crucial in identifying the functional connectivity among regions subserving language control.

## Supporting Information

Results S1 Statistical results for whole brain analysis on the switch versus L1 Chinese condition.
(PDF)

Results S2 Statistical results for whole brain analysis on the switch versus L2 English condition.
(PDF)

Results S3 Statistical results for whole brain analysis on the switch versus no naming baseline condition.
(PDF)
