# Supplementary material for: Bilingual Cognitive Control in Language Switching: An fMRI Study of English-Chinese Late Bilinguals
Source: PLoS One. 2014 Sep 2;9(9):e106468. doi: 10.1371/journal.pone.0106468 (PMC4152243; doi:10.1371/journal.pone.0106468)
Supplement: Results S1 — Statistical results for whole brain analysis on the switch versus L1 Chinese condition. (PDF) [file pone.0106468.s001.pdf]

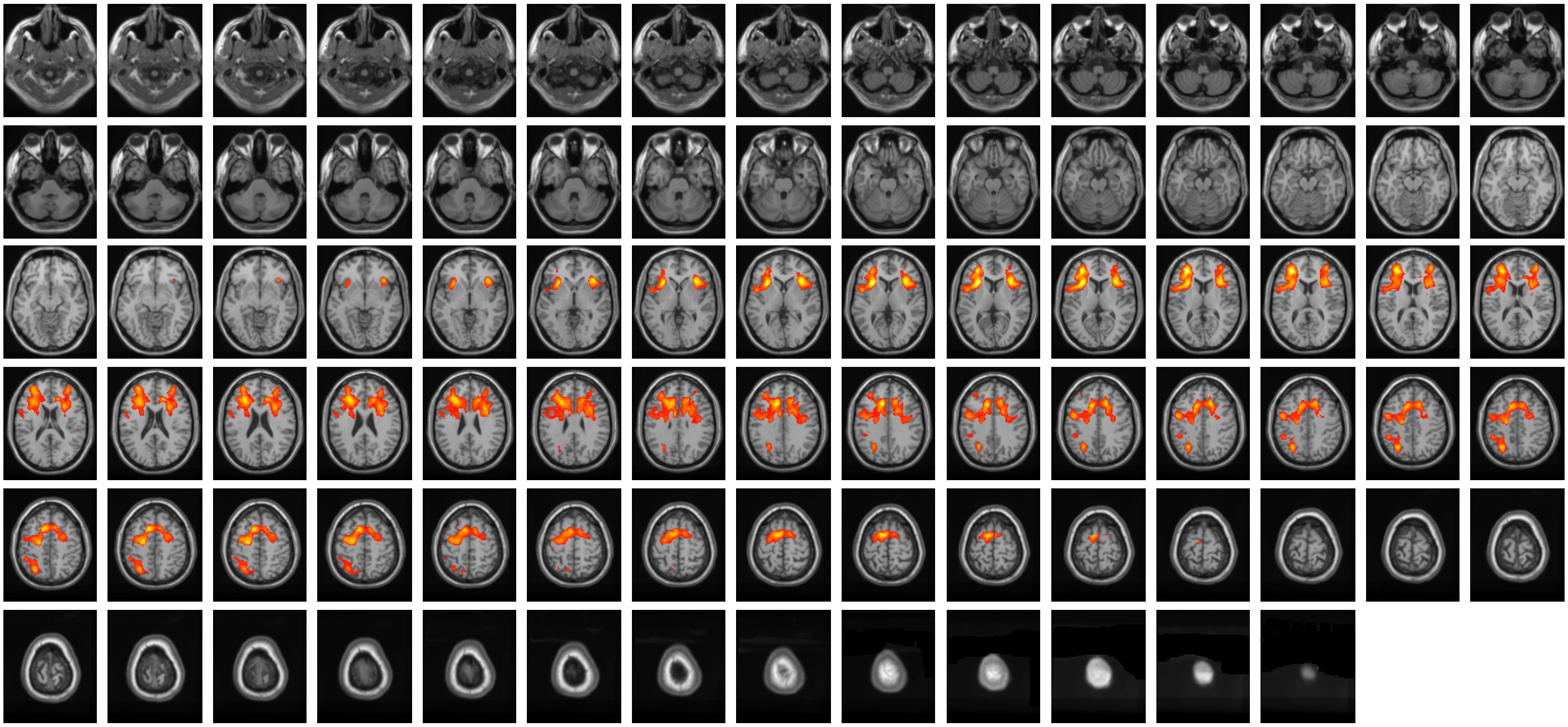

Statistics: *p-values adjusted for search volume (whole brain mask)*

set-level		cluster-level				peak-level					x,y,z {mm}		
p	c	$p_{FWE-corr}$	$q_{FDR-corr}$	k_E	p_{uncorr}	$p_{FWE-corr}$	$q_{FDR-corr}$	T	$(Z_{\equiv})$	p_{uncorr}			
**0.002**	**2**	**0.000**	**0.000**	**15396**	**0.000**	**0.000**	**0.005**	**9.37**	**5.82**	**0.000**	**-32**	**42**	**10**
						0.002	0.020	8.03	5.37	0.000	-32	18	6
						0.007	0.023	7.22	5.06	0.000	34	22	4
		**0.000**	**0.000**	**1232**	**0.000**	**0.035**	**0.036**	**6.26**	**4.65**	**0.000**	**-28**	**-64**	**46**
						0.313	0.157	4.96	3.99	0.000	-46	-44	44
						0.498	0.246	4.62	3.79	0.000	-32	-50	48

table shows 3 local maxima more than 8.0mm apart

Height threshold: T = 3.53, p = 0.001 (0.987)

Extent threshold: k = 322 voxels, p = 0.014 (0.060)

Expected voxels per cluster, <k> = 48.814

Expected number of clusters, <c> = 0.06

FWEp: 6.063, FDRp: 5.831, FWEc: 1232, FDRc: 1232

Degrees of freedom = [1.0, 21.0]

FWHM = 16.5 17.3 16.6 mm mm mm; 8.2 8.6 8.3 {voxels}

Volume: 1381472 = 172684 voxels = 271.1 resels

Voxel size: 2.0 2.0 2.0 mm mm mm; (resel = 590.61 voxels)
