# Supplementary material for: Bilingual Cognitive Control in Language Switching: An fMRI Study of English-Chinese Late Bilinguals
Source: PLoS One. 2014 Sep 2;9(9):e106468. doi: 10.1371/journal.pone.0106468 (PMC4152243; doi:10.1371/journal.pone.0106468)
Supplement: Results S2 — Statistical results for whole brain analysis on the switch versus L2 English condition. (PDF) [file pone.0106468.s002.pdf]

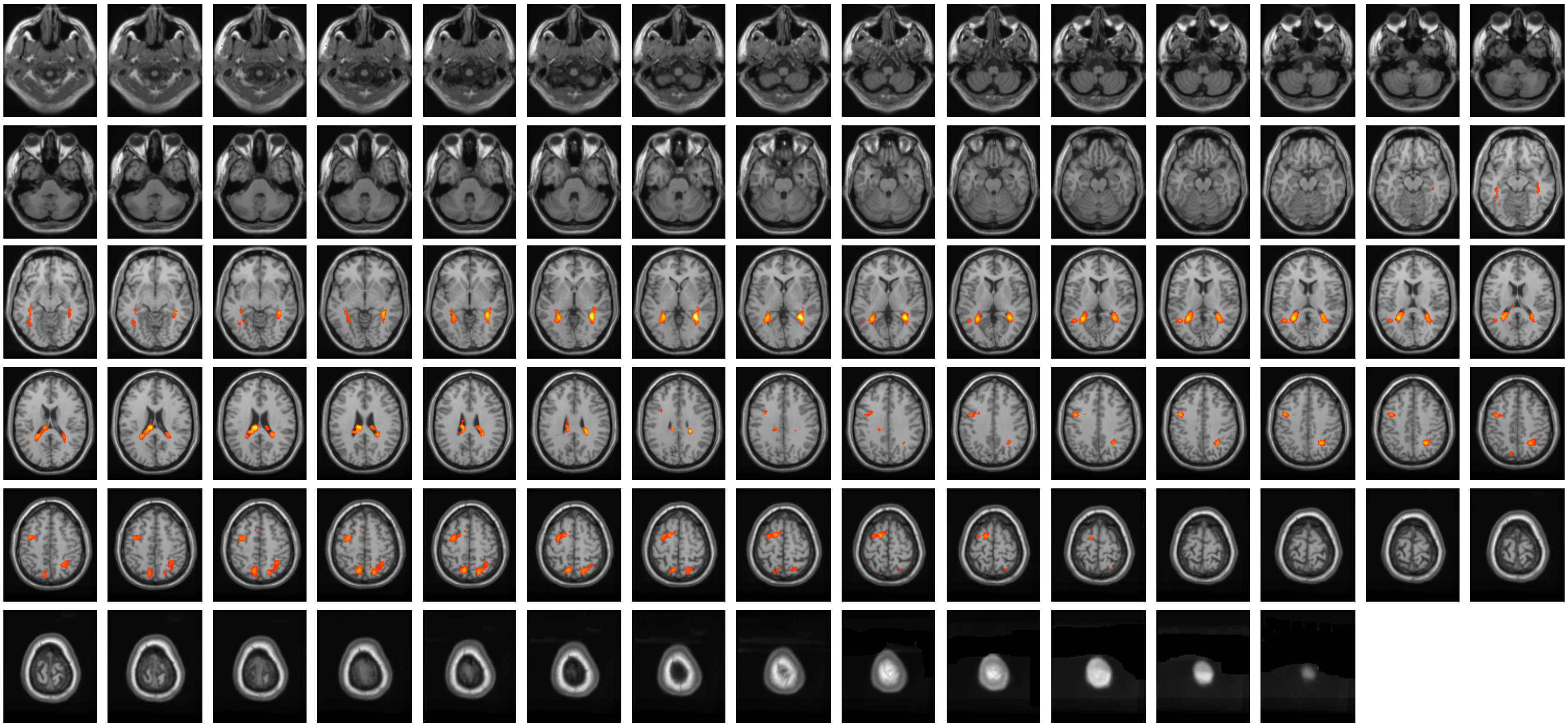

Statistics: ***p-values adjusted for search volume (whole brain mask)***

set-level		cluster-level				peak-level					
p	c	$p_{FWE-corr}$	$q_{FDR-corr}$	k_E	p_{uncorr}	$p_{FWE-corr}$	$q_{FDR-corr}$	T	$(Z_\equiv)$	p_{uncorr}	x,y,z {mm}
**0.000**	**5**	**0.000**	**0.000**	**1059**	**0.000**	**0.001**	**0.015**	**8.59**	**5.57**	**0.000**	**32 -46 2**
						0.022	0.113	6.73	4.86	0.000	22 -32 30
						0.215	0.291	5.38	4.22	0.000	26 -40 24
		**0.000**	**0.000**	**1342**	**0.000**	**0.012**	**0.097**	**7.06**	**5.00**	**0.000**	**-8 -26 22**
						0.030	0.117	6.55	4.78	0.000	-28 -50 12
						0.080	0.214	5.98	4.52	0.000	-30 -46 0
		**0.001**	**0.001**	**659**	**0.000**	**0.142**	**0.291**	**5.64**	**4.35**	**0.000**	**24 -56 42**
						0.344	0.304	5.07	4.05	0.000	18 -70 56
						0.764	0.561	4.38	3.65	0.000	32 -52 52
		**0.000**	**0.001**	**751**	**0.000**	**0.208**	**0.291**	**5.40**	**4.23**	**0.000**	**-44 0 40**
						0.255	0.304	5.27	4.16	0.000	-14 2 62
						0.442	0.363	4.89	3.95	0.000	-30 -2 56
		**0.028**	**0.027**	**322**	**0.005**	**0.212**	**0.291**	**5.39**	**4.22**	**0.000**	**-10 -68 56**
						0.838	0.602	4.24	3.57	0.000	-12 -76 48

table shows 3 local maxima more than 8.0mm apart

table shows 3 local maxima more than 8.0mm apart

Height threshold: T = 3.53, p = 0.001 (0.997)

Extent threshold: k = 322 voxels, p = 0.005 (0.028)

Expected voxels per cluster, <k> = 34.629

Expected number of clusters, <c> = 0.03

FWEp: 6.246, FDRp: 8.589, FWEc: 322, FDRc: 322

Degrees of freedom = [1.0, 21.0]

FWHM = 14.7 15.3 15.0 mm mm mm; 7.3 7.6 7.5 {voxels}

Volume: 1381472 = 172684 voxels = 382.1 resels

Voxel size: 2.0 2.0 2.0 mm mm mm; (resel = 418.98 voxels)
