# Supplementary material for: Bilingual Cognitive Control in Language Switching: An fMRI Study of English-Chinese Late Bilinguals
Source: PLoS One. 2014 Sep 2;9(9):e106468. doi: 10.1371/journal.pone.0106468 (PMC4152243; doi:10.1371/journal.pone.0106468)
Supplement: Results S3 — Statistical results for whole brain analysis on the switch versus no naming baseline condition. (PDF) [file pone.0106468.s003.pdf]

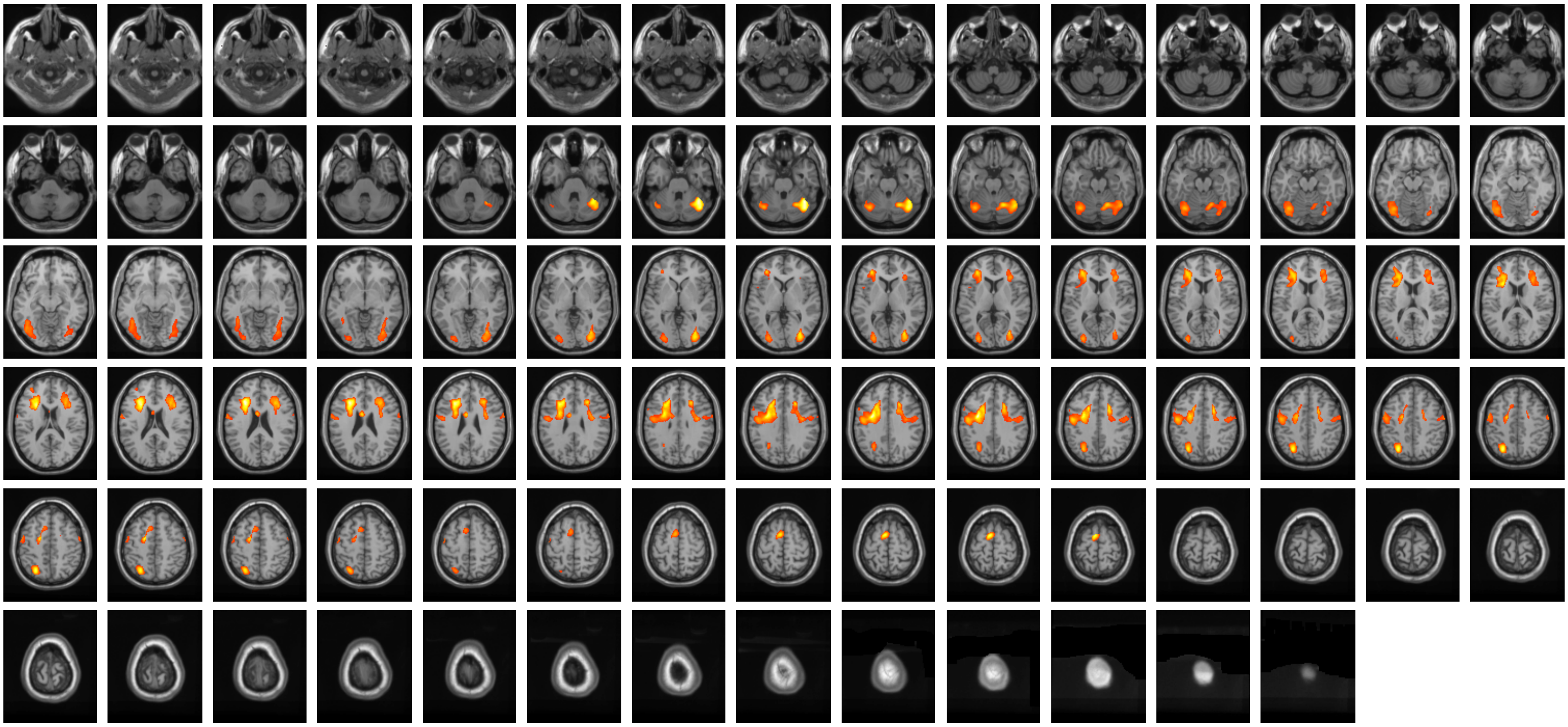

Statistics: ***p-values adjusted for search volume (whole brain mask)***

set-level		cluster-level				peak-level					x,y,z {mm}
p	c	$p_{FWE-corr}$	$q_{FDR-corr}$	k_E	p_{uncorr}	$p_{FWE-corr}$	$q_{FDR-corr}$	T	$(Z_{\equiv})$	p_{uncorr}	
**0.000**	**5**	**0.000**	**0.000**	**4403**	**0.000**	**0.001**	**0.009**	**8.36**	**5.49**	**0.000**	**-30 -4 36**
						0.002	0.009	8.06	5.38	0.000	-28 20 22
						0.013	0.034	6.89	4.93	0.000	-8 0 66
		**0.000**	**0.000**	**1939**	**0.000**	**0.002**	**0.009**	**8.10**	**5.40**	**0.000**	**40 -56 -28**
						0.003	0.011	7.77	5.28	0.000	40 -66 -28
						0.054	0.066	6.07	4.56	0.000	32 -78 0
		**0.003**	**0.001**	**663**	**0.001**	**0.010**	**0.031**	**7.05**	**5.00**	**0.000**	**-28 -68 46**
		**0.000**	**0.000**	**2126**	**0.000**	**0.056**	**0.066**	**6.06**	**4.56**	**0.000**	**20 8 38**
						0.074	0.066	5.89	4.48	0.000	26 22 28
						0.082	0.067	5.83	4.45	0.000	22 20 32
		**0.000**	**0.000**	**1538**	**0.000**	**0.179**	**0.113**	**5.37**	**4.21**	**0.000**	**-36 -68 -20**
						0.302	0.170	5.04	4.03	0.000	-30 -88 8
						0.457	0.229	4.74	3.87	0.000	-46 -74 -14

table shows 3 local maxima more than 8.0mm apart

Height threshold: T = 3.53, p = 0.001 (0.992)

Degrees of freedom = [1.0, 21.0]

Height threshold: T = 3.53, p = 0.001 (0.992)

Extent threshold: k = 663 voxels, p = 0.001 (0.003)

Expected voxels per cluster, <k> = 43.810

Expected number of clusters, <c> = 0.00

FWEp: 6.120, FDRp: 6.789, FWEc: 663, FDRc: 663

Degrees of freedom = [1.0, 21.0]

FWHM = 15.7 16.5 16.3 mm mm mm; 7.9 8.3 8.2 {voxels}

Volume: 1381472 = 172684 voxels = 302.0 resels

Voxel size: 2.0 2.0 2.0 mm mm mm; (resel = 530.06 voxels)
